# Human pluripotent embryonal carcinoma NTERA2 cl.D1 cells maintain their typical morphology in an angiomyogenic medium

**DOI:** 10.1186/1477-5751-6-5

**Published:** 2007-04-18

**Authors:** Pedro D Simões, Teresa Ramos

**Affiliations:** 1 Instituto de Tecnologia Biomédica, Laboratório de Biomateriais, Faculdade de Medicina Dentária da Universidade de Lisboa, Cidade Universitária, 1649-003 Lisbon, Portugal

## Abstract

**Background:**

Pluripotent embryonal carcinomas are good potential models, to study, *"in vitro*," the mechanisms that control differentiation during embryogenesis. The NTERA2cl.D1 (NT2/D1) cell line is a well known system of ectodermal differentiation. Retinoic acid (RA) induces a dorsal pattern of differentiation (essentially neurons) and bone morphogenetic protein (BMP) or hexamethylenebisacetamide (HMBA) induces a more ventral (epidermal) pattern of differentiation. However, whether these human cells could give rise to mesoderm derivatives as their counterpart in mouse remained elusive. We analyzed the morphological characteristics and transcriptional activation of genes pertinent in cardiac muscle and endothelium differentiation, during the growth of NT2/D1 cells in an inductive angiomyogenic medium with or without Bone Morphogenetic Protein 2 (BMP2).

**Results:**

Our experiments showed that NT2/D1 maintains their typical actin organization in angiomyogenic medium. Although the beta myosin heavy chain gene was never detected, all the other 15 genes analyzed maintained their expression throughout the time course of the experiment. Among them were early and late cardiac, endothelial, neuronal and teratocarcinoma genes.

**Conclusion:**

Our results suggest that despite the NT2/D1 cells natural tendency to differentiate into neuroectodermal lineages, they can activate genes of mesodermal lineages. Therefore, we believe that these pluripotent cells might still be a good model to study biological development of mesodermal derivatives, provided the right culture conditions are met.

## Background

Teratocarcinomas are highly malignant tumours, cells that have a big potential to differentiate in other cell types. They are considered to recapitulate many events occurring in early embryogenesis, but with a lesser degree of organization and regulation, being also called embryonal carcinomas (EC) [1-5]. The "undifferentiated elements" of these tumors are composed of EC cells or malignant pluripotent stem cells that appear, in culture, as embryoid body-like structures. After differentiation, they can also be histologically positive for many somatic tissues such as bone, muscle, nerve and others, these constituting the "differentiated elements" of these tumors or the teratoma components [6].

The NT2/D1 cell line is a human EC that does not require any feeder layer to preserve its undifferentiated potential and it may form embryoid body-like structures *in vitro*, a feature also observed in other EC tumours [7-9]. The presence of some differentiating agents as RA, in the culture medium, seems to activate the neuroectodermal differentiating program [10], where the NT2/D1 cells give rise to neural cells but also nonneural cells. However, even without this agent, NT2/D1 can originate some neuronal-like phenotypes [11].

When NT2/D1 cells give rise to neurons less than 10% of the cells adopt this phenotype, while the rest of the cells in the nonneural populations remain to be further analyzed and identified. Thus the hypothesis of mesodermal derivatives from NT2/D1 cannot be excluded [10,12,13]. Nonneural cells were suggested to be primarily epithelial in nature, by immunocytochemical staining, but the exact identity of such cells has never been documented [11]. It is suggested that this cell line although expressing mesodermal genes, does not adopt a mesodermal differentiation program [13]. Most authors agree that this cell line does not give rise to mesodermal derivatives; however, opposite evidence has been recently documented, in a BMP2 induced differentiation [14].

We believe that NT2/D1 might be the human counterpart of P19, a mouse teratocarcinoma cell line with cardiac differentiation potential. One of the genes highly expressed in P19, that disappears after the differentiation is finished, is *Cripto *[15]. This gene codes for a small cysteine-rich protein that contains an epidermal growth factor (EGF)-like motif and a CRIPTO/FRL1/Cryptic (CFC) motif. CRIPTO is anchored in the cell membrane by a glycosyl-phosphatidylinositol linkage where it functions as a coreceptor for Nodal, a member of the TGF-β family [16]. It can be detected in the trophoblast and in the inner cell mass of mouse blastocysts, becoming, thereafter restricted to developing myocardium. *Cripto *knockout is lethal in mouse and its inactivation *in vivo *results in the loss of detection of some cardiac transcripts, such as, *alpha myosin heavy chain (α-MyHC)*, *beta myosin heavy chain (β-MyHC)*, *atrial myosin light chain 2 (MLC-2a)*, *ventricular myosin light chain 2 (MLC-2v) *and *atrial natriuretic factor ANP *[17]. In embryonic stem cells (ESCs), its loss does not affect cell commitment to the mesenchymal, endodermal or ectodermal lineage, but results also in the absence of transcription of cardiac specific genes. For all this, it has been suggested, recently, that *CRIPTO *is a master gene regulator in cardiomyogenesis. Expression of *CRIPTO *by NT2/D1 cells has been shown by Baldassarre *et al*. [18].

To find a human embryonic cell line that could differentiate with high frequency into myocardium and to identify the factor(s) that would lead the cells through that path, would be a powerful tool to study cardiomyogenesis, *in vitro*.

Here, we have studied the cell morphology and the transcription profile of the NT2/D1 cells cultured for 30 days with and without BMP2. The gene expression analyzed was particularly focused on EC, endothelial, neural progenitors and early and late cardiac differentiation genes. An angiomyogenic medium was tested as differentiation culture medium.

## Results

### Morphological analysis

**Figure 1:**
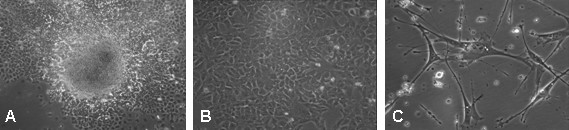
**NT2/D1 cells in expansion medium**. Phase contrast images of three typical morphologies of NT2/D1 cells in expansion medium, (RPMI, FBS and antibiotics) analyzed in a CK2 Olympus microscope: A. Embryoid body-like structures, magnification 40×; B. epithelioid-like phenotype, magnification 100×; C. neuronal-like phenotype, magnification 200×.

The typical NT2/D1 morphological characteristics, as the increase in nuclear/cytoplasm volume ratio, the flattening of the cells, the prominent nucleoli (Figure 1), the polymerized actin organization (phalloidin staining) and ultrastructural analysis were used as the morphological criteria to monitor cell differentiation.

**Figure 2:**
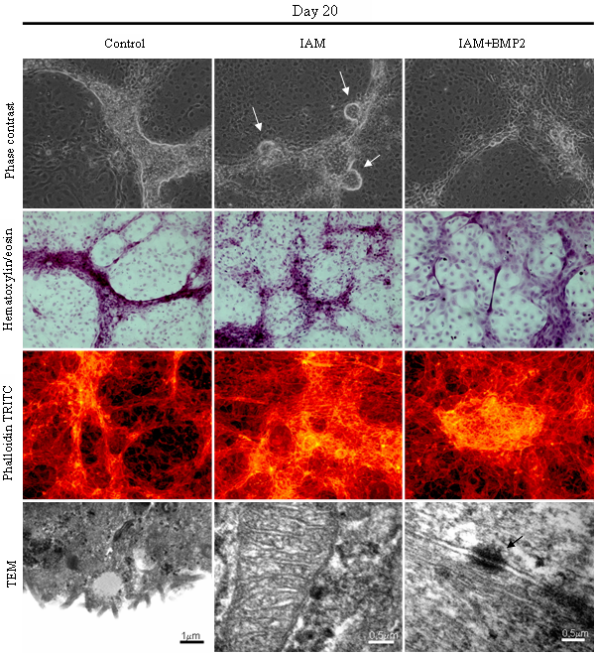
**NT2/D1 cells in IAM medium**. Cell culture images of NT2/D1 cells after 20 days growing in control, IAM and IAM+BMP2 medium. All the non fluorescent and fluorescent images are 100× magnified and analyzed in a CK2 and BX50 Olympus microscope, respectively. Ultrastructural images have a scale bar in the bottom right corner. NT2/D1 cells formed many embryonic body-like structures (white arrows), surrounded by an epithelioid squamous layer of adherent flattened cells. Desmosomes can be seen between adjacent cells (TEM black arrow). To see non fluorescent and fluorescent images of days 1, 5, 10 and 30, please [see Additional file 4].

As can be seen in figure 2, after 20 days in culture, in control as well as in IAM medium, NT2/D1 cells can form many embryonic (white arrows) body-like structures, surrounded by an epithelioid squamous layer of adherent flattened cells, resembling a visceral endoderm phenotype [19] with the interesting fact of presenting many vilosities. When in an overconfluency state, many structures present some similarities with an endothelial angiogenic network. In contrast, neuronal-like cells could not be seen, indicating a possible inhibition of that phenotype with 20% FBS or in IAM. Also, no muscular structures were ever detected during the time course of the experiment.

### Ultrastructural analysis

As mentioned above, some villosities could be detected in TEM (figure 2, TEM, control). This finding is consistent with the tendency that the NT2/D1 cells have to differentiate through an epithelial path. Desmosomes are visible between adjacent cells (figure 2, black arrow), which would be consistent with a muscular phenotype, however, specific morphologies of the latter tissue, like sarcomeres, were not ever found, [see Additional file 1].

### Transcriptional analysis

**Figure 3:**
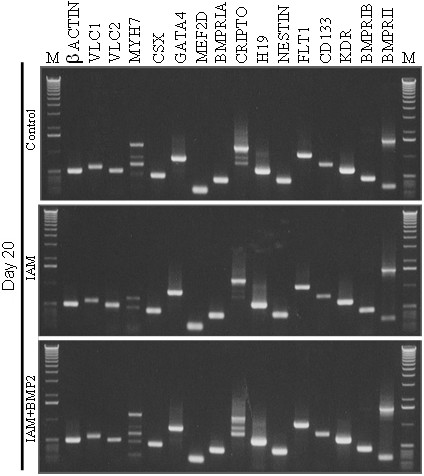
**Expression studies**. Polaroid images of 0,8% agarose gels with Ethidium Bromide of polymerase amplified gene products of NT2/D1 cells cDNA. In the first and last lanes one can see the 1 Kb ladder (Invitrogen). To see gel images of the days 1, 5, 10 and 30, or other details related to the amplified products and primers, please [see Additional file 4].

To see if would be any tendency of NT2/D1 to differentiate in a more muscle related or endothelial related fashion, genes specific for that tissues were chosen, ([see Additional file 2] and Figure 3):

#### Muscle related genes

At all time points analyzed, NT2/D1 cells expressed undifferentiated cardiac specific genes, like *GATA4*, *CSX1 *and *MEF2D*, as well as differentiated cardiac specific genes as *VLC1 *and *VLC2*. The *slow muscle myosin heavy chain gene (MYH7)*, found in cardiac but also in some skeletal muscles, was the sole gene, of those analyzed that was not detect in this study. Although the primers for the *MYH7 *were specific for this gene, the bands that were found were nonspecific products of the polymerase reaction, giving rise to a specific pattern of expression in the particular cell line tested. Curiously, non muscular tissues also gave rise to nonspecific products with the specific *MYH7 *primers, [see Additional file 3].

#### Endothelial progenitor related genes

Transcripts of *CD133 *and the *VEGF receptor 2, KDR*, could be detected at all time points of the experiment. This finding could reflect the angiogenic potential of these cells [20,21]. In fact, there are some images in the differentiation cultures that remind angiogenic like networks, but further studies are obviously needed to prove this concept.

#### Other progenitor related genes

Knowing that NT2/D1 expresses the *CD133 *and that these cells easily undergo neural differentiation, we would expect to detect expression of *NESTIN *[22,23]. That was indeed the case. *NESTIN *expression was detected from the first day of the experiment. On the other hand, the expression of *FLT1 *indicated that these cells could be considered not only endothelial but also haematopoietic progenitor cells as described by others [24,25].

#### Bone morphogenetic protein receptors (BMPRs)

Because one of our main hypothesis was that BMP2 was an inducer of mesodermal differentiation in NT2/D1 cultures, we analyzed whether the main BMP receptor genes, namely *BMPRIA*, *BMPRIB *and *BMPRII *[26,27] were transcriptionally active. Type II receptors can bind ligands in the absence of type I receptors, but they need their respective type I receptors to transduce the signal [28,29]. *BMPRIA*, *BMPRIB *and *BMPRII *transcripts could be detected in the 5 time points of the experiment. Interestingly, the heavy isoform of *BMPRIB *was only detected after 30 days in the angiomyogenic medium. The mechanism underlying this phenomenon remains to bee clarified.

#### NT2/D1 related genes

*H19 *is a gene that is expressed in a vast number of organs during fetal development. In adults, however, expression is confined to lung, thymus, skeletal and cardiac muscle [30]. Accordingly, in EC cells it is expressed after induction of differentiation [15,30-33]. Our experiments showed that NT2/D1 expressed *H19 *throughout the time course of the experiment, [see Additional file 4].

As mentioned before, one of the genes highly expressed in P19 cells, a murine EC, with cardiac differentiation potential is *Cripto*, that disappears after the differentiation is finished [15]. As expected, we detect in the NT2/D1 cell line relative good levels of *CRIPTO *expression that changed to a more unspecific pattern in the last days of the experiment, suggesting that NT2/D1 had differentiated [18]. The expected band for *CRIPTO *was 1199 bp. So, the significance of the several unexpected bands that have appeared in NT2/D1 after some days in culture remains to be clarified, [see Additional file 4]. This similarity between P19 and NT2/D1 cells supported our initial hypothesis, that in the appropriate conditions, NT2/D1 cells could give rise to mesodermal derivatives, such as skeletal or cardiac muscle.

## Discussion

Despite the difference of human and mouse EC at the level of cell surface markers, they share some common characteristics, such as, the morphology of little cytoplasm, prominent nucleoli and the preferential clustering growth pattern. In this line of thought, one can say that the mouse teratocarcinoma cell line, P19, has a relevant similar behavior to NT2/D1, also, regarding the potential of both to originate neuroectodermal derivatives in the presence of retinoic acid.

However, in the presence of RA P19 cells can still give rise to mesodermal derivatives, such as, smooth muscle, skeletal muscle and cardiac muscle [34,35]. The last two processes, can be also induced by dimethylsulphoxide (DMSO) [36] and the cardiac differentiation inhibited by noggin [37], a BMP2 antagonist, indicates that probably, this growth factor is essential in this inductive process.

BMP2, belonging to the transforming growth factor superfamily, is involved in many important processes in early human and mouse development [19,38-43] and have a variety of effects in human and mouse teratocarcinomas. It can induce epithelial differentiation in NT2/D1 [44] and endodermal differentiation in GCT27X-1 EC and human ESCs [19,45], however in P19 cells it can give rise also to mesodermal derivatives like cardiac muscle [37]. Contrarily to human ESCs, treatment of mouse ESCs with BMP2 and/or FGF2 efficiently enhanced cardiomyogenesis [46].

For this, BMP2 has been considered to be an inhibitor of the neural differentiation program and an embryonic ectoderm [11] or extra-embryonic endoderm epidermal inducer in human pluripotent stem cells [19], but also a mesodermal inducer in the mouse pluripotent stem cells [47]. Only recently, has the latter role been documented [14], in humans.

We also believe that BMP2 can indirectly induce NT2/D1 cells into mesodermal derivatives like cardiac muscle, because: 1) observations from amphibians, chicks and more recently from mice, have proposed that the factors produced by embryonic endoderm are good candidates to be cardiogenic inducers [48]; 2) BMPs, as BMP2, have a role in visceral endoderm differentiation in the mouse [49] and in the human ESCs system [50]; 3) the mouse P19CL6noggin EC cell line that overexpresses the BMP2 antagonist noggin and has lost it potential to differentiate into cardiomyocytes, can regain that property when in the presence of excess BMP2 protein [37]; 4) recently, it has been shown that the mouse visceral endoderm-like cell line END2, derived from P19 EC, when cocultured with the parental cells [51] or mouse ESCs [52] induces spontaneous aggregation and differentiation into cardiac muscle, in the absence of BMP2. The same effect could be obtained with END2 conditioned medium [52]. Curiously, in humans one can also obtain cardiomyocytes by coculturing human ESCs with mouse END2 [52,53]. The latter findings indicate that the visceral endoderm-like cells and not the BMP2, are the primary key for myocardial differentiation. Apparently, END2 cells produce one or more factors that promote cardiac differentiation.

Hence, one can say that the main potential of P19 cells to spontaneously differentiate into cardiac derivatives may come from the factors produced by the cells that undergone the visceral endoderm-like differentiation, thus, inducing other cells to the mesodermal lineage. So, we speculate that when the BMP2 is present it may contribute indirectly to cardiomyogenesis by increasing the number of visceral endoderm like cells in culture.

Then, if BMP2 can inhibit the neural fate and induce the mesodermal and the visceral endoderm-like phenotype in NT2/D1 cells it would possibly induce some cells to the cardiac lineage too. Unfortunately, morphological signals of muscle differentiation as myotube formation and sarcomeric intracellular actin organization were not detected, in an angiomyogenic inductive medium, in the presence of BMP2 or in 5-Aza treated cells, as we did not see any muscle beating areas at all the time points of the experiment, indicating that the NT2/D1 were not committed to mesodermal lineages like striated muscle, but instead, they differentiate mainly into the expected epithelioid morphologies. There were some cultures that exhibit some angiomyogenic like structures, but further studies are needed to confirm that.

However, transcripts from the cardiomyogenic (*VLC1*, *VLC2*, *MEF2D*, *GATA4 *and *CSX1*) and endothelial (*KDR *and *CD133*) mesodermal lineages, bone morphogenetic protein receptors (*BMPRIA*, *BMPRIB *and *BMPRII*) and NT2/D1 specific were detected in a qualitative and constitutive manner during the time course of the experiment.

*CRIPTO*, a suggested master gene regulator in cardiogenesis, is one of the genes highly expressed in cardiomyogenic P19 cells. Accordingly, we detect in the NT2/D1 cell line good levels of *CRIPTO *expression, strengthening our initial hypothesis, that in appropriate conditions, NT2/D1 cells can possibly give rise to mesodermal derivatives, such as cardiac muscle. During the time course of the experiment, *CRIPTO *transcripts tended to become more difficult to visualize while *H19 *transcripts more easy to detect, a signal that NT2/D1 were differentiating, at least regarding this genes, in a similar manner as their mouse counterpart P19.

## Conclusion

We believe that the apparent blockage of the differentiation pathways *in vitro *through a mesodermal commitment may reside on unknown pos-transcriptional or pos-translational mechanisms.

Another possibility is that the aneuploid state of NT2/D1 cells inhibits the key differentiating genes, and that what was once an advantage, regarding the tumor clonal selection of differentiation-resistant cells, is now a relevant obstacle in differentiation studies like this one.

Still, identifying the factors capable of regulating differentiation of pluripotent human EC cell lines, will give us clues for as to how manipulate human stem cells, an important raw material for *in vitro *generation of tissues for transplantation therapy.

## Methods

### Cell culture

NT2/D1 cells (Stratagene) were cultured and expanded in 80 cm^2 ^flasks in RPMI 1640 medium without Glutamine (Invitrogen), with 10 mM HEPES (Invitrogen), 20 μM 2-β-Mercaptoetanol (Sigma), 2 mM Glutamine (Invitrogen) 10% FBS (Invitrogen) and 1% (v/v) Penicillin 10000 IU/ml + Streptomycin 10 mg/ml (Invitrogen) and left for growth to 80% confluence, harvested by scraping and reseeded 1:4, continuously until they were needed.

In angiomyogenic induction, NT2/D1 cells were suspended in inductive angiomyogenic medium (IAM) with endothelial cell growth supplement (ECGS) as described by Shmelkov *et al *[54]: Briefly, cells were cultured in M199 Medium (Invitrogen) supplemented with 20% FBS (Invitrogen), 10 mM HEPES (Invitrogen), 1 U Heparin (Sigma), 1% (v/v) Penicillin 10000 IU/ml + Streptomycin 10 mg/ml (Invitrogen), 10 ng/ml VEGF (Sigma), 5 ng/ml bFGF (Sigma), 50 ng/ml BDNF (Invitrogen) and 150 μg/ml ECGS and seeded at 1000 cells/cm^2 ^on glass or thermanox coverslips coated with 2% gelatin, in 24-well dishes. The seeded cells were left for the first 24 h in 10 μM 5-Azacytidine and then washed and resuspended in the same initial medium. BMP2, at the concentration of 100 ng/ml, was added to IAM (IAM+BMP2). Medium without VEGF, bFGF, BDNF and ECGS was used as control (Control). Cells were harvested on day 1, 5, 10, 20 and 30 after initiation of culture and used for histological, ultrastructural and RT-PCR analysis.

### Reverse Transcription Polymerase Chain Reaction (RT-PCR) Analysis

Total RNA was extracted according to Trizol total RNA extraction protocol (Invitrogen). cDNA first strand synthesis was performed on 1 μg total RNA, by using the ThermoScript RT-PCR System (Invitrogen) with an oligo dT primer. As a second step, cDNA samples were subjected to PCR amplification, in a separate tube, using primers specific for the gene of interest, [see Additional files 5 and 6], with the Platinum Taq DNA Polymerase High Fidelity (Invitrogen) using 40 cycles at 56°C. For each gene, the DNA primers were derived from different exons, allowing the distinction between cDNA and contaminating genomic DNA amplifications. Primers were synthesized for the following human genes: *ACTB*, *VLC1*, *VLC2*, *MYH7*, *CSX1*, *GATA4*, *MEF2D*, *BMPRIA*, *CRIPTO*, *H19*, *Nestin*, *FLT1*, *CD133*, *KDR*, *BMPRIB*, and *BMPRII*.

### Histochemistry

Glass coverslips, containing the NT2/D1 cells, were fixed for 30 min with the addition of buffered formaldehyde pH 7.0 into the cell culture wells at a 2% final concentration, washed in D-PBS pH 7,2–7,4 (Invitrogen) and stored at 4°C until processed. When processed, coverslips were washed in water, immersed in Harris's hematoxylin (Merck) for 10 minutes, washed in water, differentiated in 70% EtOH with 1% HCl for 10 seconds, immersed in eosin Y (Merck) for 10 seconds and washed again. Stained cells in coverslips were dehydrated in graded series of ethanol, followed by xylol (Merck), and mounted on glass microscope slides, cell side down, using entellan (Merck).

### Phalloidin staining

Dilution of phalloidin-TRITC stock solution (Sigma) and washing of the glass coverslips were performed in D-PBS pH 7,2–7,4 containing 0,05% saponin (Sigma). Coverslips were fixed for 30 min with the addition of buffered formaldehyde pH 7.0 into the cell culture wells at a 2% final concentration, incubated with 1 μg/ml phalloidin-TRITC solution for 90 min at RT and washed 3 times. The coverslips were then mounted on microscope slides, cell side down, using Glicerol:D-PBS (1:1) and visualized in a Olympus BX50 microscope.

### Ultrastructural analysis

Thermanox coverslips (Nunc) from day 20, were fixed in 0,1 M sodium cacodylate buffer (EMS) pH 7,3 with 3% gluteraldeyde (EMS) for 1 h, washed in 0,1 M sodium cacodylate, followed by 1% osmium tetroxide (EMS) in the same buffer for 1 h and washed again, first in cacodylate buffer and then in 0,1 M sodium acetate buffer (Merck) pH 5,0. Fixed cells were incubated with 1% uranyl acetate (EMS) in 0,1 M sodium acetate buffer pH 5,0 for 1 h, washed in the same buffer, dehydrated in graded series of ethanol, followed by propylene oxide (EMS), embedded in epon (EMS) and left for 3 days at 70°C. Thin sections cut on a LKB III microtome with a diamond knife for transmission electron microscopy (TEM) were stained on 300 mesh square copper grids with 2% uranyl acetate and lead citrate (Fluka) and observed in a Philips CN10 transmission electron microscope. All the steps were made at room temperature, except when otherwise documented.

### Sequencing

The PCR products were purified using Qiaquick PCR purification kit (Quiagen) as described by the manufacturer and visualized by ethidium bromide after electrophoresis in a 1% agarose gel. A molecular weight standard a 1 kb DNA ladder (Invitrogen,) was used. All sequences were determined in an automated DNA capillary sequencer CEQ 2000-XL (Beckman Coulter, USA, in ICAT – Lisbon Faculty of Sciences Sequencing Services) by a dye-labeled dideoxy termination method (DTCS, Dye Terminator Cycle sequencer start kit, Beckman Coulter). Two sequencing reactions were performed using the two primers used for PCR amplification. The sequences obtained, were compared with existing gene data from the National Centre for Biotechnology Information (GenBank) for all the amplified genes. All the primers have given rise to the expected amplified fragments.

## Authors' contributions

PDS conceived the study, carried out all the experimental procedures of the current work and drafted the manuscript. TR participated in the elaboration of the design of the study and has made substantial contributions to the analysis, interpretation of data and critically revising of the manuscript. All the authors have read and approved the final manuscript.

## Supplementary Material

Additional File 1 Human pluripotent embryonal carcinoma NT2/D1 in an angiomyogenic medium. TEM images at differentiation day 20. Click here for file

Additional File 2 **Human pluripotent embryonal carcinoma NT2/D1 in an angiomyogenic medium. RT-PCR Qualitative analysis**. This is not a quantitative analysis, what is the best choice to compare transcripts of different genes. However, a qualitative analysis can be done, comparing the intensity of the gel bands with the intensity of a house keeping gene, in our case the β actin. The scores we have chosen to construct these graphics were: 0 – PCR product not detected; 1 – PCR product with much less intensity of the β actin product; 2 – PCR product with less intensity of the β actin product; 3 – PCR product with the intensity of the β actin product; 4 – PCR product with more intensity of the β actin product; 5 – PCR product with much more intensity of the β actin product. Of course, that with this method the conclusions one can take are very limited, and only big differences have caught our attention. The main observations were: i) contrarily to what we have expected, in general, all genes were equally expressed in the different time points analyzed, with the exception of the *MYH7 *that was never detected; ii) the gel band of *CRIPTO *has tendency to change to a specific pattern during NT2/D1 differentiation; iii) a DNA band of *BMPRIB *appears in late stages of NT2/D1 differentiation. We cannot exclude DNA contamination in the PCR reaction, but if so, and because all the other reactions used the same cDNA, why was this gene the only one presenting a DNA amplified band? We do not have a explanation for this. iv) the heavy band of *BMPRII *appears only in late stages of NT2/D1 differentiation. Click here for file

Additional File 3 **Human pluripotent embryonal carcinoma NT2/D1 in an angiomyogenic medium. The case of the *MYH7 *PCR**. The *MYH7 *primers were constructed and positively verified for accuracy using cDNA samples of human masseter and human left ventricle, which are muscle tissues known to express this gene in a high relative level. With that samples we could obtain single band PCRs positively verified by sequencing. (see the two last lanes before the ladder in the gel image). The first time we have used the *MHY7 *primers in non muscle tissues, we have obtained a pattern of expression that was very interesting at the beginning, because we thought that we were leading with an alternative splicing event. However, after sequencing, the PCR products of different sizes that the NT2/D1 and other samples gave rise in the polymerase reaction with the *MYH7 *primers, had nothing to do with the expected results, as one can see in the figure. The five non specific bands in the NT2/D1 lane are marked from 1 to 5 with the respective sequentiation results shown below. Click here for file

Additional File 4 **Human pluripotent embryonal carcinoma NT2/D1 in an angiomyogenic medium. 100× images**. Phase contrast, hystochemical and fluorescent images of NT2/D1 cells at time points 1, 5, 10, 20 and 30 days in Control, IAM and IAM+BMP2 culture mediums. Transcriptional analysis of 16 genes at the same time points is also shown. Click here for file

Additional File 5 Diagram of the NT2/D1 Transcripts Click here for file

Additional File 6 Primer data and sizes of the PCR amplified products Click here for file
