# Supplementary figures and images for: Human pluripotent embryonal carcinoma NTERA2 cl.D1 cells maintain their typical morphology in an angiomyogenic medium
Source: J Negat Results Biomed. 2007 Apr 18;6:5. doi: 10.1186/1477-5751-6-5 (PMC1863432; doi:10.1186/1477-5751-6-5)

**
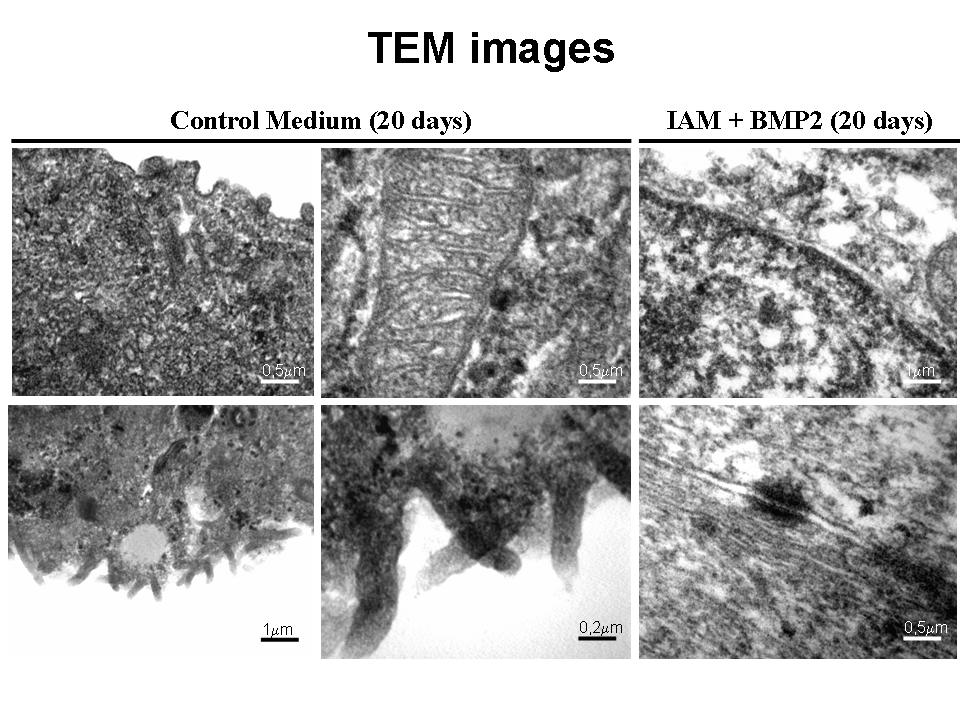
**

**Additional file 1**

**Human pluripotent embryonal carcinoma NT2/D1 in an angiomyogenic medium**

Supplement: Additional File 1 — Human pluripotent embryonal carcinoma NT2/D1 in an angiomyogenic medium. TEM images at differentiation day 20. [file 1477-5751-6-5-S1.doc]

**Additional file 2**

**Human pluripotent embryonal carcinoma NT2/D1 in an angiomyogenic medium**


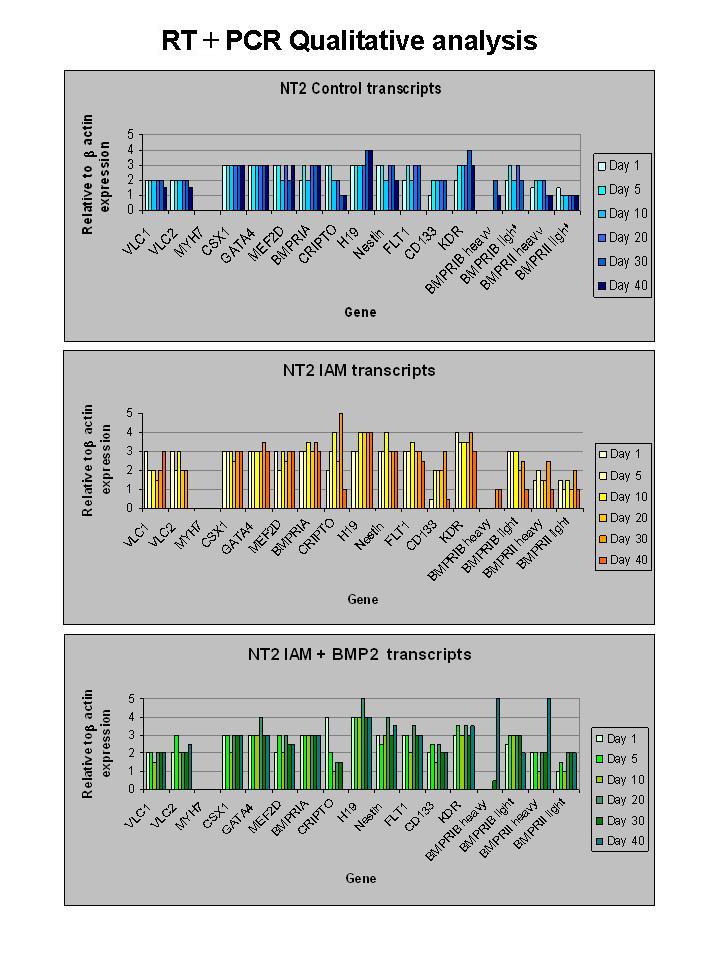

Supplement: Additional File 2 — Human pluripotent embryonal carcinoma NT2/D1 in an angiomyogenic medium. RT-PCR Qualitative analysis. This is not a quantitative analysis, what is the best choice to compare transcripts of different genes. However, a qualitative analysis can be done, comparing the intensity of the gel bands with the intensity of a house keeping gene, in our case the β actin. The scores we have chosen to construct these graphics were: 0 – PCR product not detected; 1 – PCR product with much less intensity of the β actin product; 2 – PCR product with less intensity of the β actin product; 3 – PCR product with the intensity of the β actin product; 4 – PCR product with more intensity of the β actin product; 5 – PCR product with much more intensity of the β actin product. Of course, that with this method the conclusions one can take are very limited, and only big differences have caught our attention. The main observations were: i) contrarily to what we have expected, in general, all genes were equally expressed in the different time points analyzed, with the exception of the MYH7 that was never detected; ii) the gel band of CRIPTO has tendency to change to a specific pattern during NT2/D1 differentiation; iii) a DNA band of BMPRIB appears in late stages of NT2/D1 differentiation. We cannot exclude DNA contamination in the PCR reaction, but if so, and because all the other reactions used the same cDNA, why was this gene the only one presenting a DNA amplified band? We do not have a explanation for this. iv) the heavy band of BMPRII appears only in late stages of NT2/D1 differentiation. [file 1477-5751-6-5-S2.doc]

**
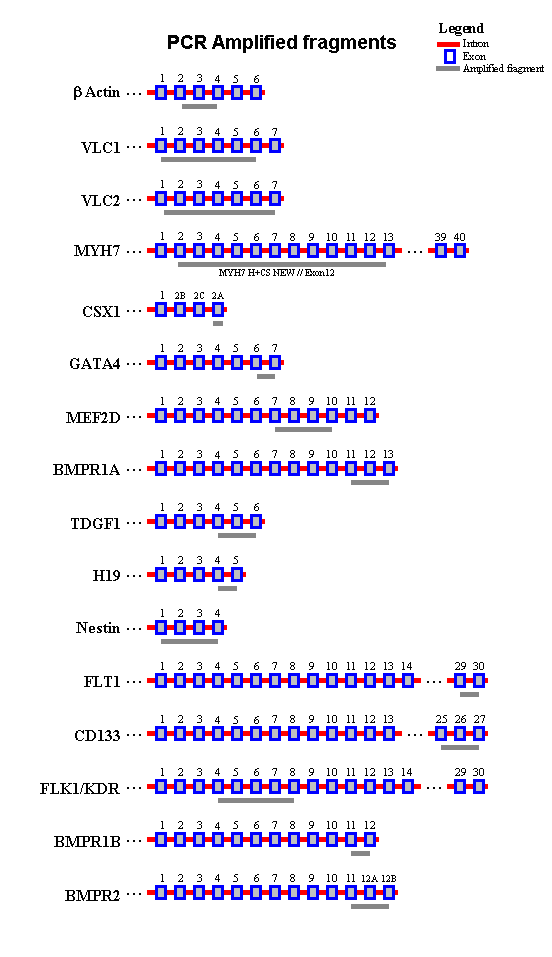
**

**Additional file 5**

**Diagram of the NT2/D1 Transcripts**

Supplement: Additional File 5 — Diagram of the NT2/D1 Transcripts [file 1477-5751-6-5-S5.doc]
