# Supplementary material for: Human pluripotent embryonal carcinoma NTERA2 cl.D1 cells maintain their typical morphology in an angiomyogenic medium
Source: J Negat Results Biomed. 2007 Apr 18;6:5. doi: 10.1186/1477-5751-6-5 (PMC1863432; doi:10.1186/1477-5751-6-5)
Supplement: Additional File 3 — Human pluripotent embryonal carcinoma NT2/D1 in an angiomyogenic medium. The case of the MYH7 PCR. The MYH7 primers were constructed and positively verified for accuracy using cDNA samples of human masseter and human left ventricle, which are muscle tissues known to express this gene in a high relative level. With that samples we could obtain single band PCRs positively verified by sequencing. (see the two last lanes before the ladder in the gel image). The first time we have used the MHY7 primers in non muscle tissues, we have obtained a pattern of expression that was very interesting at the beginning, because we thought that we were leading with an alternative splicing event. However, after sequencing, the PCR products of different sizes that the NT2/D1 and other samples gave rise in the polymerase reaction with the MYH7 primers, had nothing to do with the expected results, as one can see in the figure. The five non specific bands in the NT2/D1 lane are marked from 1 to 5 with the respective sequentiation results shown below. [file 1477-5751-6-5-S3.doc]

**Additional file 3**

**Human pluripotent embryonal carcinoma NT2/D1 in an**

**angiomyogenic medium. The case of the MYH7 PCR**


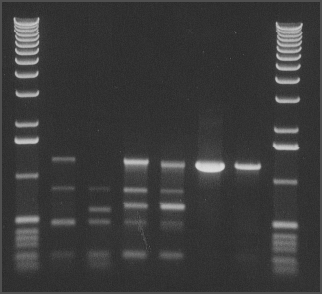


1 Kb Ladder

UCB

Hyppocampus

RH30

NT2

Left Ventricle

Masseter

1 Kb Ladder

**1**

**2**

**3**

**4**

**5**

| **Band** | **Gene** | **Chr** |
| --- | --- | --- |
| **1** | Tubulin Tyrosin Ligase Like 9 (TTLL9) intron | 20 |
| **2** | Repeat elements (MIR, AluSx, L2) | 8 |
| **3** | Fatty Acid Desaturase 1(FADS1) | 11 |
| **4** | Unknown region | 2 |
| **5** | Not sequenced | - |
