# Supplementary material for: Human pluripotent embryonal carcinoma NTERA2 cl.D1 cells maintain their typical morphology in an angiomyogenic medium
Source: J Negat Results Biomed. 2007 Apr 18;6:5. doi: 10.1186/1477-5751-6-5 (PMC1863432; doi:10.1186/1477-5751-6-5)
Supplement: Additional File 4 — Human pluripotent embryonal carcinoma NT2/D1 in an angiomyogenic medium. 100× images. Phase contrast, hystochemical and fluorescent images of NT2/D1 cells at time points 1, 5, 10, 20 and 30 days in Control, IAM and IAM+BMP2 culture mediums. Transcriptional analysis of 16 genes at the same time points is also shown. [file 1477-5751-6-5-S4.doc]

**
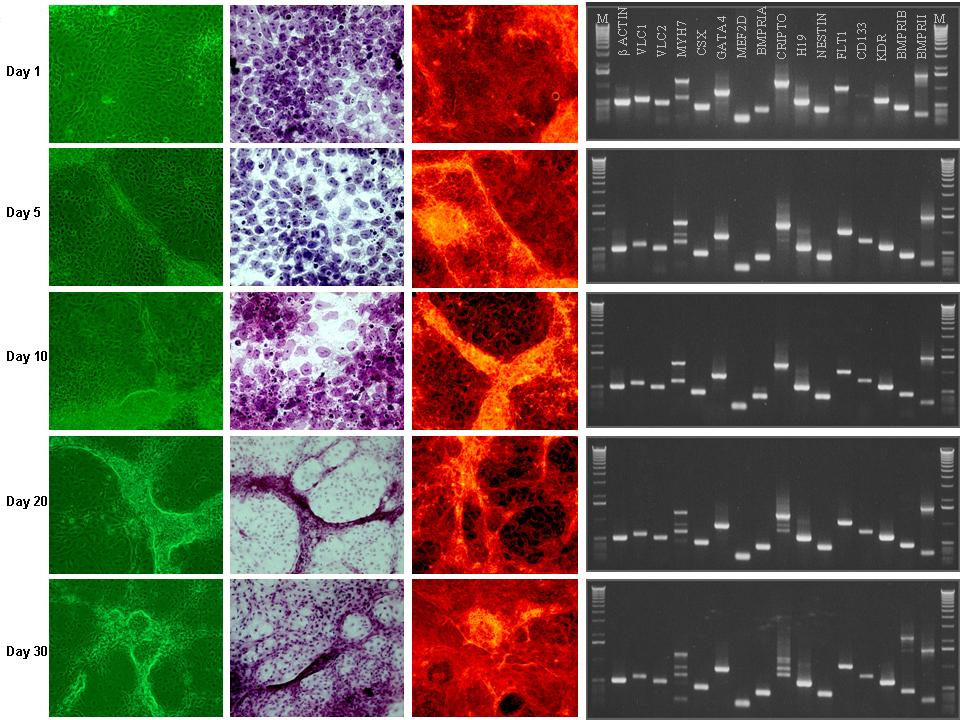
**

**Phase contrast**

**Hematoxylin/Eosin**

**Phalloidin**

**RT + PCR**

**Control**

**Additional file 4**

**Human pluripotent embryonal carcinoma NT2/D1 in an angiomyogenic medium.**

**(100X images)**


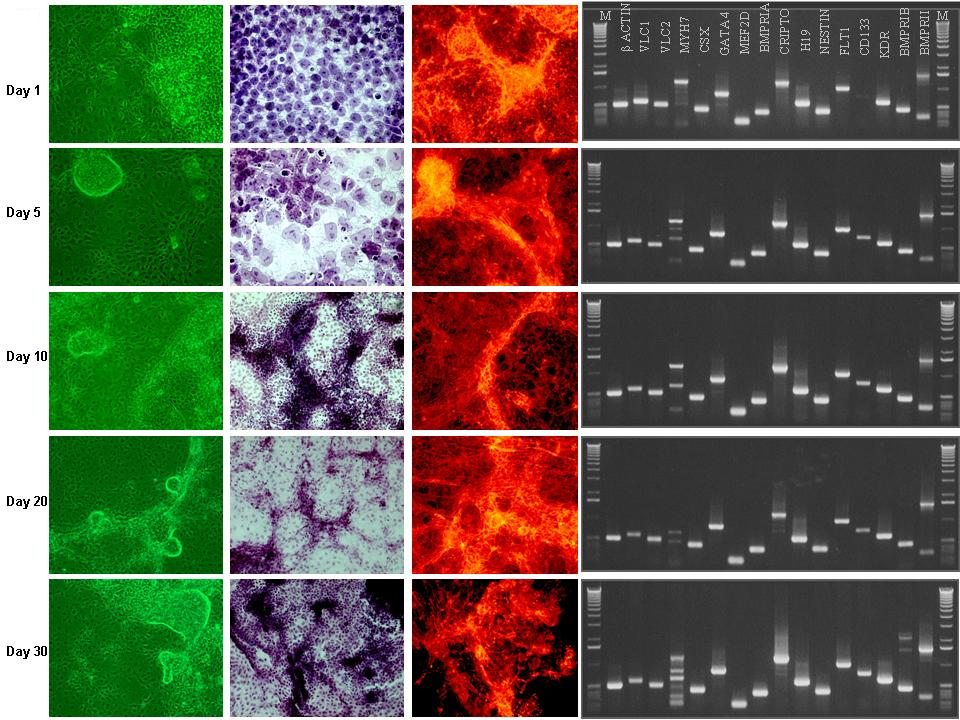


**Phase contrast**

**Hematoxylin/Eosin**

**Phalloidin**

**RT + PCR**

**IAM**


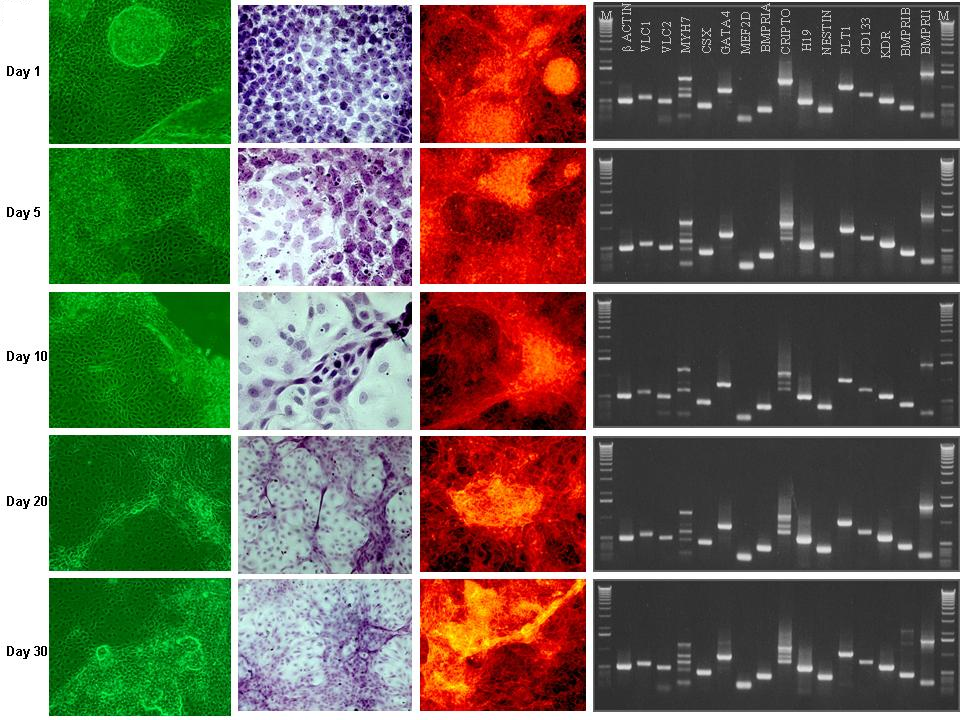


**Phase contrast**

**Hematoxylin/Eosin**

**Phalloidin**

**RT + PCR**

**IAM+BMP2**
