# Supplementary material for: Human pluripotent embryonal carcinoma NTERA2 cl.D1 cells maintain their typical morphology in an angiomyogenic medium
Source: J Negat Results Biomed. 2007 Apr 18;6:5. doi: 10.1186/1477-5751-6-5 (PMC1863432; doi:10.1186/1477-5751-6-5)
Supplement: Additional File 6 — Primer data and sizes of the PCR amplified products [file 1477-5751-6-5-S6.doc]

**Additional file 6 - Primer data and PCR amplified products**

|  | **Expected** | **product** | **Gene** |  |  |  |  | **PCR fragment** |
| --- | --- | --- | --- | --- | --- | --- | --- | --- |
| **Gene** | **cDNA (bp)** | **gDNA (bp)** | **Bank** | **5´ primer** | **nt** | **3´primer** | **nt** | **sequenciation** |
| **Control** |  |  |  |  |  |  |  |  |
| β - actin | 513 | 1080 | M10277.1 | 5’ ATA TCg CCg CgC TCg TCg TC 3’ | (1103-22) | 5’ Tgg CAT ggg ggA ggg CAT AC 3’ | (2169-88) | **OK** |
| **Muscle specific** |  |  |  |  |  |  |  |  |
| VLC1 | 591 | 5150 | NT_022517.17 | 5’ ATg gCC CCC AAA AAg CCA gAg 3’ | (52-72) | 5’ ggT TTA gCT ggA CAT gAT gTg 3’ | (5181-201) | **OK** |
| VLC2 | 505 | 9397 | L01652.1 | 5’ ATg gCA CCT AAg AAA gCA AAg 3’ | (1272/4)-(2591/2609) | 5’ CCC CTC CTA gTC CTT CTC TTC 3’ | (10648-68) | **OK** |
| MYH7 | 1216 | 4915 | M57965.1 | 5´ CCT Tgg CCC CTT TCC TCA TCT gT 3’ | (5206-28) | 5’ CAT gAg gTA ggC AgA CTT gT 3’ | (94558-77) | **OK** |
| Csx/Nkx2.5 ^1^ | 418 | 418 | NC_000005.7 | 5´ TAT AAC gCC TAC CCC gCC TAT 3’ | (662-42) | 5’ TAA TCg CCg CCA CAA ACT CTC C 3’ | (266-45) | **OK** |
| GATA4 | 823 | 2032 | NC_000008.8 | 5’ ggC CTg TCA TCT CAC TAC gg 3’ | (52799-818) | 5’ TgA TgA ggC TgT gCT gTg gT 3’ | (54811-30) | **OK** |
| MEF2D ^2^ | 185/206 | 8046 | NC_000001.7 | 5’ CAg gAA Agg ggT TAA TgC ATC AC 3’ | (13311-289) | 5’ ggA gAg CTC TgC ACT ggT CAA CTg 3’ | (5289-66) | **OK** |
| BMPRIA | 369 | 2136 | NC000010.8 | 5’ gTg ggC ACC AAA CgC TAC AT 3’ | (164919-38) | 5’ CAT CTT ggC AAg CgT CTT CTT A 3’ | (00-00) | **OK** |
| **NTERA2 specific** |  |  |  |  |  |  |  |  |
| TDGF1 | 1199 | 2376 | M96955.1 | 5’ TgT gCC TgC CCT CCC TCC TT 3’ | (4305-24) | 5’ ggA AAC TTg CCC TTC CAT TTA gCC 3’ | (6657-80) | **OK** |
| H19 ^3^ | 575 | 655 | M32053.1 | 5’ TAC AAC CAC TgC ACT ACC T 3’ | (2754-72) | 5’ Tgg AAT gCT TgA Agg CTg CT 3’ | (3389-408) | **OK** |
| Nestin ^4^ | 389 | 3466 | NC_000001.7 | 5’ CAg CgT Tgg AAC AgA ggT Tgg 3’ | (7784-64) | 5’ Tgg CAC Agg TgT CTC AAg ggT Ag 3’ | (4341-19) | **OK** |
| FLT1 | 1010 | 4319 | NC_000013.8 | 5’ ACC CAA ggC CTC gCT CAA gA 3’ | (188395-414) | 5’ ACA AAT CAA AAC ATg CCA CgA ATg 3’ | (191690-192713) | **OK** |
| CD133 | 762 | 10748 | NC_000004.8 | 5’ TCA TAA AgA TCA TgT ATA Tgg TAT TC 3’ | (11206-181) | 5’ TTg TCA gAT ggA gTT ACg CA 3’ | (478-59) | **OK** |
| **Endothelial specific** |  |  |  |  |  |  |  |  |
| CD133 | 762 | 10748 | NC_000004.8 | 5’ TCA TAA AgA TCA TgT ATA Tgg TAT TC 3’ | (11206-181) | 5’ TTg TCA gAT ggA gTT ACg CA 3’ | (478-59) | **OK** |
| FLK1/KDR | 630 | 4655 | NC_000004.9 | 5’ gTg ACC AAC ATg gAg TCg Tg 3’ | (10215-34) | 5’ CCA gAg ATT CCA TgC CAC TT 3’ | (14850-69) | **OK** |
| **Brain specific** |  |  |  |  |  |  |  |  |
| BMPRIB | 453 | 2229 | NC_000004.9 | 5’ gTg CCC AgT gAC CCC TCT TA 3’ | (276852-71) | 5’ ACA AgT TAC CCA AgC ggT TTC T 3’ | (279059-80) | **OK** |
| **Others** |  |  |  |  |  |  |  |  |
| BMPRII | 297/1577 | 7104 | NC_000002.9 | 5’ gAT gCA gAg gCT Cgg CTT AC 3’ | (175886-905) | 5’ TTg gAA TgA ACT gCC CTg TTA C 3’ | (182968-89) | **OK** |

**References**

1. Kodama, H., Hirotani, T., Suzuki, Y., Ogawa, S. & Yamazaki, K. Cardiomyogenic differentiation in cardiac myxoma expressing lineage-specific transcription factors. Am J Pathol 161, 381-9 (2002).

2. Breitbart, R. E. et al. A fourth human MEF2 transcription factor, hMEF2D, is an early marker of the myogenic lineage. Development 118, 1095-106 (1993).

3. Fukuzawa, R. et al. High frequency of inactivation of the imprinted H19 gene in "sporadic" hepatoblastoma. Int J Cancer 82, 490-7 (1999).

4. Kukekov, V. G. et al. Multipotent stem/progenitor cells with similar properties arise from two neurogenic regions of adult human brain. Exp Neurol 156, 333-44 (1999).
